# GoActive: a protocol for the mixed methods process evaluation of a school-based physical activity promotion programme for 13–14year old adolescents

**DOI:** 10.1186/s13063-018-2661-0

**Published:** 2018-05-21

**Authors:** Stephanie T. Jong, Helen Elizabeth Brown, Caroline H. D. Croxson, Paul Wilkinson, Kirsten L. Corder, Esther M. F. van Sluijs

**Affiliations:** 1 MRC Epidemiology Unit and UKCRC Centre for Diet and Activity Research (CEDAR), University of Cambridge School of Clinical Medicine, Box 285, Institute of Metabolic Science, Cambridge Biomedical Campus, Cambridge, CB2 0QQ UK; 2 Nuffield Department of Primary Care Health Sciences, University of Oxford, Radcliffe Primary Care Building, Radcliffe Observatory Quarter, Woodstock Rd, Oxford, OX2 6GG UK; 3 Department of Psychiatry, University of Cambridge and Cambridgeshire and Peterborough NHS Foundation Trust, Cambridge, UK

**Keywords:** Process evaluation, Complex intervention, Mixed methods, Physical activity, Behaviour change, Adolescent health, Randomized controlled trial, Protocol

## Abstract

**Background:**

Process evaluations are critical for interpreting and understanding outcome trial results. By understanding how interventions function across different settings, process evaluations have the capacity to inform future dissemination of interventions. The complexity of Get others Active (GoActive), a 12-week, school-based physical activity intervention implemented in eight schools, highlights the need to investigate how implementation is achieved across a variety of school settings. This paper describes the mixed methods GoActive process evaluation protocol that is embedded within the outcome evaluation. In this detailed process evaluation protocol, we describe the flexible and pragmatic methods that will be used for capturing the process evaluation data.

**Methods:**

A mixed methods design will be used for the process evaluation, including quantitative data collected in both the control and intervention arms of the GoActive trial, and qualitative data collected in the intervention arm. Data collection methods will include purposively sampled, semi-structured interviews and focus group interviews, direct observation, and participant questionnaires (completed by students, teachers, older adolescent mentors, and local authority-funded facilitators). Data will be analysed thematically within and across datasets. Overall synthesis of findings will address the process of GoActive implementation, and through which this process affects outcomes, with careful attention to the context of the school environment.

**Discussion:**

This process evaluation will explore the experience of participating in GoActive from the perspectives of key groups, providing a greater understanding of the acceptability and process of implementation of the intervention across the eight intervention schools. This will allow for appraisal of the intervention’s conceptual base, inform potential dissemination, and help optimise post-trial sustainability. The process evaluation will also assist in contextualising the trial effectiveness results with respect to how the intervention may or may not have worked and, if it was found to be effective, what might be required for it to be sustained in the ‘real world’. Furthermore, it will offer suggestions for the development and implementation of future initiatives to promote physical activity within schools.

**Trial registration:**

ISRCTN, ISRCTN31583496. Registered on 18 February 2014.

**Electronic supplementary material:**

The online version of this article (10.1186/s13063-018-2661-0) contains supplementary material, which is available to authorized users.

## Background

Process evaluations are critical for interpreting and understanding outcome trial results [1], and have the capacity to inform future dissemination of interventions by understanding how interventions function across different settings. In particular, Grant et al. [2] highlight the need to critically examine the delivery of each component and the processes, or underlying mechanisms, of any complex intervention. The UK Medical Research Council’s (MRC) framework for designing and evaluating complex interventions recommends conducting a process evaluation in order to assess fidelity and quality of implementation, clarify causal mechanisms and identify contextual factors associated with variation in outcomes [3]. By conducting a process evaluation, researchers can examine the barriers and facilitators that influence the delivery of the intervention within different contexts, investigating successes and failures of implementation in order to maximise learning from the trial delivery of an intervention [4].

Previously published process evaluation protocols identify the importance of pre-specifying methods when conducting process evaluation [4–9]. Grant et al. [6] consider it ‘best practice’ to publish process evaluation protocols, and recognize the importance of doing so to improve the standards of trials. Similarly, Ellard et al. [7] acknowledge the importance of process evaluations, and advocate for process evaluations to be held to the same standard as the main outcome evaluation by publishing the protocol. Despite increasing emphasis on the importance of process evaluation of complex interventions, explicit reporting of process evaluations and publishing of process evaluation protocols is limited [2]. Recent studies in the field of school-based physical activity interventions have combined qualitative and quantitative methods for process evaluations [10–13]. Of these, we identified only one published peer-reviewed protocol [9].

Following recommendations, and the outline of previously published process evaluation protocols, this detailed protocol extends the brief description of the mixed-methods process evaluation for the Get Others Active intervention (GoActive) published as part of the main trial protocol [14] (10/07/2017; version 4). By conforming to set standards, the paper will pre-specify the methods used in the GoActive process evaluation. This paper will first briefly describe the GoActive intervention being evaluated, prior to detailing the process evaluation-specific research questions, design, and methods.

Whilst the GoActive trial began in 2016, process evaluation data had not been released at the time of writing.

### The GoActive intervention

**Fig. 1:**
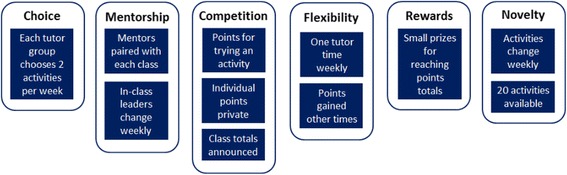
GoActive six key tenets

GoActive is a physical activity promotion programme targeting English Year 9 students (aged 13–14 years). The intervention and its effect evaluation have been described in detail previously [14, 15]. GoActive uses specific behaviour-change techniques (e.g. goal setting behaviour, social support, rewards, and role modelling), depicted as the six key tenets in Fig. 1, to increase adolescents’ daily moderate-to-vigorous physical activity (MVPA). Each tutor group (class or home room class) chooses two activities per week from a selection provided. Teachers of Year 9 tutor groups within intervention schools are encouraged to use one tutor time weekly to do one of the chosen activities. There are 20 GoActive activities available, using little or no equipment, appealing to a wide variety of students (including Ultimate Frisbee, Zumba and Hula Hoop). Materials to support delivery of these activities are available on a password-protected website, which includes a short video introducing each activity and activity instructions (Quick Cards). These offer an overview of each activity, a short explanation and suggestions for adaptations, and provide advice, safety tips and ‘factoids’.

**Fig. 2:**
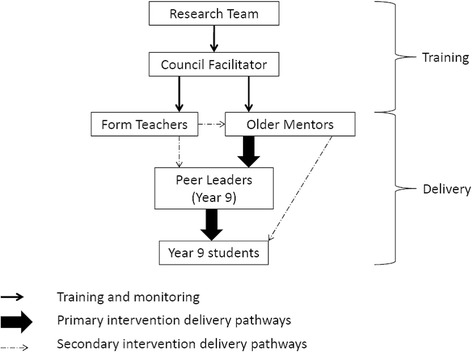
Overview of leadership structure

GoActive is implemented using a tiered-leadership system (Fig. 2), where mentors (older adolescents within the school) and in-class Year 9 peer leaders encourage Year 9 students to try activities each week. Over the course of 12 weeks, mentors remain paired with a Year 9 tutor group for the duration of the intervention, whereas the peer leaders (two per tutor group each week, one male and one female) change every week. Additionally, a local authority-funded intervention facilitator engages with the intervention in two phases: (1) facilitated support and ( 2) distant support. During the first 6 weeks of delivery the facilitator provides active facilitated support at weekly meetings with mentors, and provides distant support thereafter.

Students gain points for trying the GoActive activities, at any time, in or out of school. Crucially, points are awarded for engagement, not for duration or intensity. Each student can log their individual points on the password-protected GoActive website. An individual’s points remain private on the GoActive website, but points are accumulated as a tutor group to create inter-tutor group competition (within schools), with graphs available to view via the website. Students can claim small rewards upon reaching individual point thresholds (i.e. a sports bag (15 points), t-shirt (50 points), or hoodie (150 points)). After the 12-week intervention period, each intervention school holds an award ceremony where additional prizes (e.g. Frisbees, and drink bottles) are provided to Year 9 students identified by tutor group teachers as ‘Most Improved’ (one male and one female). One male and one female student are awarded ‘Most Engaged’ (identified by GoActive website points), and an award is presented to the most engaged mentor (£40 vouchers) identified by the contact teacher, or tutor group teachers.

### The GoActive effectiveness evaluation

The overall aim of the GoActive effectiveness evaluation is to assess the 10-month effectiveness of the GoActive intervention in increasing average daily objectively measured MVPA among 13–14-year-old adolescents. GoActive is evaluated in a cluster randomised controlled trial, including 16 schools across Cambridgeshire and Essex, UK, and 2858 Year 9 students at baseline. The main outcome is measured with accelerometry. Secondary outcomes include time spent sedentary; time-specific time spent in activity intensities; self-reported physical activity and psychosocial outcomes; cost-effectiveness and cost-utility analyses; and anthropometric data (height, weight, waist circumference, and body fat percentage). Outcomes will be assessed at baseline (T1; Sept-Dec 2016), interim (week 6) (T2; Mar-Apr 2017), post intervention (week 14–16) (T3; May-Jul 2017), and at 10 month follow up (main outcome, T4; Mar-Jul 2018). See published protocol for further details on the quantitative outcome evaluation [14].

### The GoActive process evaluation aim and objectives

The main aim of the GoActive process evaluation is to understand what worked and why in the implementation of the GoActive intervention, and to contribute to the interpretation of the findings of the effectiveness evaluation results. The objectives of the GoActive process evaluation are therefore:

1. To assess the reach, dose and fidelity of intervention delivery; to document how the intervention was implemented, and ascertain whether the intervention’s essential functions (elements of the intervention) were adapted to suit individual settings
2. To explore the GoActive intervention from the perspective of Year 9 students, mentors, teachers, and facilitators, to describe participants’ views of the intervention (including intervention acceptance)
3. To consider the maintenance and sustainability of the intervention and, if proven effective, the possible dissemination of the GoActive intervention

The process evaluation is designed to observe the implementation of GoActive and will not be used to intervene with how a school implements the GoActive intervention where deviations from the intervention protocol are detected.

The design of the process evaluation was informed by the MRC guidance on the process evaluation of complex interventions [3, 16, 17]. The MRC identify three essential features of understanding the processes through which outcomes are achieved: context, implementation and mechanisms of impact. The application of this framework is described as follows:

- **Context:** An examination of the broader school culture (e.g. school structure, school leadership team, school focus) into which the GoActive intervention is introduced, and how it may have influenced and interacted with the delivery and functioning of the intervention's essential functions.
- **Implementation:** An examination of how the GoActive intervention is delivered. The structure, resources, and processes by which the intervention is achieved, the extent to which the intervention was delivered as intended, and any adaptations made to the intervention.
- **Mechanisms of impact:** An examination of the processes through which the GoActive intervention affects outcomes through understanding how participants (Year 9 s, mentors, teachers, and facilitators) respond to and interact with the intervention, and how the intervention supports change (or not).

Underpinned by the MRC’s guidelines, six process evaluation components will be assessed: fidelity, dose delivered and received, reach, recruitment, and context [18]. The MRC’s guidelines explicitly align with these process evaluation components, as well as the objectives that will be achieved (Table 1).

**Table 1:** The MRC’s essential features aligned with the study’s process evaluation components, and objectives

MRC’s three essential features	Process evaluation components	Objective
Implementation	Fidelity	1
	Dose delivered	1
Mechanisms of impact	Dose received	2
	Reach	2
	Recruitment	2
Context	Context	3

### The GoActive logic model

**Fig. 3:**
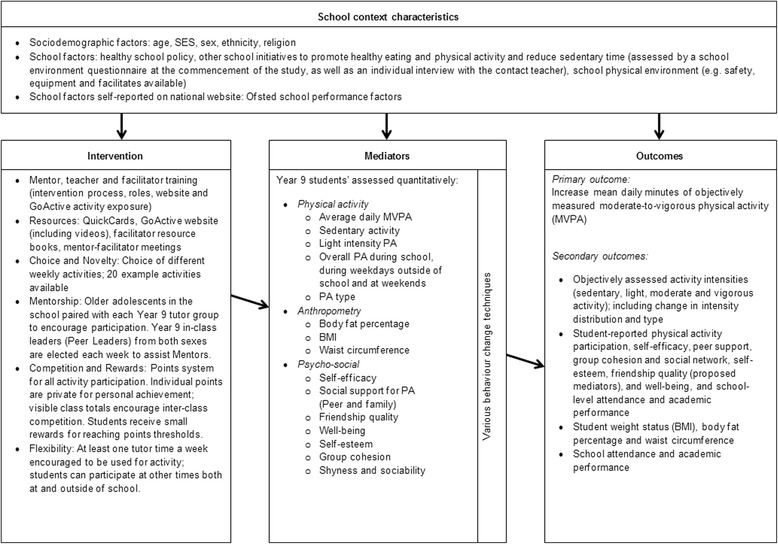
The GoActive logic model

The logic model for GoActive attempts to visually represent the processes of the intervention, and the outcomes it aims to achieve. The school contexts in which the intervention is delivered, and any potential impact this may have on the intervention is considered, as well as the content of the intervention, and the theoretical underpinnings. The logic model for the study is shown in Fig. 3.

## Methods

### Overall design of process evaluation

The mixed methods process evaluation, embedded in the main GoActive trial, will be led by a mixed methods researcher. A number of qualitative and quantitative methods will be employed per participant group (outlined in Table 2). Process evaluation quantitative data collection will occur in both the control and intervention arms of the trial, whereas qualitative data will only be generated through methods conducted in the intervention arm of the trial.

**Table 2:** Methods for data collection with participants

	Questionnaire (intervention and control)	Observation (intervention only)	Web analytics (intervention only)	Focus group interviews (intervention only)	Individual interviews (intervention only)
Year 9 students	✓	✓	✓	✓	✓
Mentors	✓	✓	✓	✓	
Form group teachers	✓				
Contact Teacher					✓
Facilitators	✓		✓		✓
Year 9 tutor groups		✓			

Within intervention evaluation research, mixed methods designs are considered useful in the assessment of acceptability, feasibility, and processes [19]. A mixed methods design will allow the research team to build a comprehensive profile of each school and its implementation of the intervention, to examine differences across subgroups (e.g. Year 9 s, mentors and teachers), and across qualitative and quantitative datasets. Both types of data, (i.e. qualitative and quantitative), will be collected during the same phase of the research and merged during analysis and interpretation [19]. However, in a sequential step quantitative data from T1 will be used to identify the sample of Year 9 students included in T3 qualitative data collection, following which analyses will be merged and interactive [19].

Ethical approval for the process evaluation was obtained from the University of Cambridge Psychology Research Ethics Committee PRE.2015.126. Parental consent for Year 9 students, and assent from students themselves, was sought prior to T1 measurements. Mentors and contact teachers will provide written consent or assent (for those younger than 16 years, for whom parental consent will also be obtained) to participate in process.

**Fig. 4:**
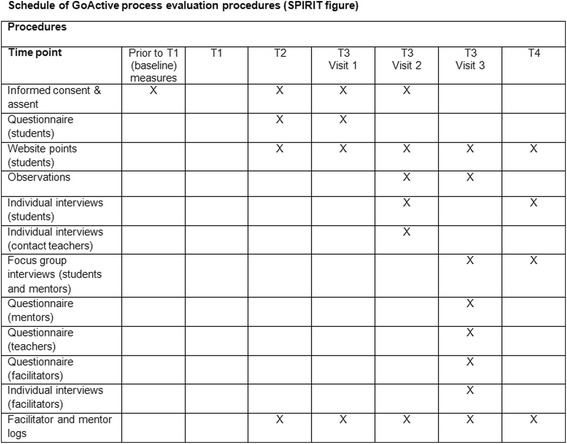
Schedule of GoActive process evaluation procedures (SPIRIT figure)

The process evaluation protocol was developed according to the Standardized Protocol Items: Recommendations for Interventional Trials (SPIRIT) guidelines (see Additional file 1 and Fig. 4) [20].

All data will be collected and managed in line with International Conference on Harmonisation Good Clinical Practice guidelines. Data will be stored securely at the MRC Epidemiology Unit, University of Cambridge, UK.

### Quantitative data collection

Quantitative data will be collected through participant questionnaires, as well as via website analytics of points logged and pages visited within the GoActive website. A process evaluation section will be included in the outcome questionnaires administered at T2 and T3 for all Year 9 students, both in the control and intervention arms of the trial. It is anticipated that having control participants complete a process evaluation questionnaire will determine possible contamination [21], and will provide a baseline measure to ameliorate a social desirability response. Separate process evaluation questionnaires will be prepared for mentors, tutor group teachers in the intervention arm of the trial, and the local authority-funded facilitators.

#### Participant questionnaires

##### Year 9 students

The process evaluation section of the T2 questionnaire will be delivered to all Year 9 students participating in the trial, gathering information about the regularity and consistency of GoActive sessions, and their experiences of the intervention. For example, students will respond to statements about the GoActive intervention: ‘The GoActive study...is fun’, ‘…introduces me to new activities’, ‘…made me step out of my comfort zone’. Four response categories will be provided on a strongly agree to strongly disagree Likert scale, with a fifth option for students who perceivably ‘did not take part’ in the intervention. Perceived competence of the mentors, perceived role of the tutor group teachers, and the extent to which participants used the GoActive resources (e.g. website usage, QuickCards) will also be assessed. The process evaluation section of the T3 questionnaire will repeat most of the T2 questions, with some additional questions. The T3 questionnaire will follow up on any changes occurring since the previous time point, and will act as a post-intervention participant questionnaire. Students from both intervention and control schools will complete the T4 (10-month follow-up; Mar-Jul 2018) process evaluation questionnaire. This will contain questions on the extent to which participants are still engaged with GoActive after the facilitated intervention has ended, and what potential facilitators and barriers may have contributed to maintenance or decline in intervention participation.

##### Mentors, teachers, and facilitators

All older peer mentors, intervention school tutor group teachers, and facilitators will be provided with a process evaluation questionnaire at T3. These questionnaires will gather key sociodemographic characteristics of participants (e.g. age, sex), information about the frequency of GoActive sessions, experiences of the GoActive intervention (including training components), and sustainability of the intervention.

#### Website analytics

Website data will be collected from Year 9s entering individual points through the GoActive website (a point per activity completed), as well as website usage. Point-based data, as well as the type of activity a participant received points for, will be collected for every individual participant who entered points on the GoActive website. The website usage data will also include the number of user logins, as well as logs of the pages visited within the GoActive website, for example, video explanations of activities or resources such as QuickCards.

### Qualitative data collection

Qualitative data will be collected through observations, focus group and individual interviews, and mentor and facilitator written logs. All observations and interviews will be carried out by the same trained mixed methods researcher. Reflection after each interview and discussion with independent researchers will facilitate the development of the interview guides (e.g. to identify any required additions, or to pursue emerging themes) (refer to Additional files 2 and 3).

#### Observations of GoActive sessions

Observations will occur in all eight intervention schools. One Year 9 class participating in GoActive from each intervention school will be nominated by the school for observation. Observations will provide a greater understanding of each school context, and will help to determine how the GoActive intervention is conducted across different school settings. The researcher will aim to observe one class on two occasions to account for researcher reactivity. Ideally, one observation will be conducted in the first six weeks of the intervention, and one after the first six weeks of the intervention to allow an observation in each of the two intervention phases (i.e. facilitated, and distant support). This will be subject to school availability and scheduling constraints. Information obtained via observations will lead to a broad understanding of the intervention fidelity and implementation, and provide insight in to mentor and Year 9 student interaction, whether instructions from mentors were delivered clearly to Year 9 students, and whether these instructions were in line with the received training. Observations will also highlight Year 9 student interactions, Year 9 student engagement with the intervention, questions asked and suggestions made from Year 9 students during the session, as well as verbal and non-verbal cues that demonstrated engagement with the intervention.

Observations will be non-participative in nature, with the researcher watching and taking field notes from a distance [22]. The researcher will take necessary steps to minimise the intrusion, “not overplaying the ‘status’ card, making contact with the teacher beforehand, clarifying the purpose and likely outcome of the observation…to be as natural and unstaged as possible” (p. 16) [23]. Following Creswell’s [22] series of observational steps, the researcher will design an observational protocol as a method for recording notes at the school. Descriptive and reflective notes about the implementation of the GoActive intervention will be included in the protocol, along with notes on the physical setting within the school, interactions and events and activities, the mixed methods researcher’s reactions, as well as noting what did not take place. The researcher will prepare her notes as soon as possible after the observation, providing a “rich narrative description of the people and events under observation” (p. 168) [22].

#### Field notes

Field notes will include any reflections recorded immediately after the visit, completed by the researcher. Changes in the delivery of the intervention (including the process of training, facilitator numbers, mentor numbers etc.), or notes on school implementation of the GoActive intervention will be recorded. This information will provide a recollection of any modifications to the GoActive intervention that occur, which may have had an impact on the intervention outcomes. Additional notes derived from emails, or other research team correspondence with schools or facilitators will also be recorded.

### Recruitment for interviews

Year 9 students at intervention schools will be able to indicate at T2 whether they would be willing to be contacted about participating in an interview; those who respond positively will be provided with an additional information sheet to clarify the interview procedure (both individual and focus group). Consenting students will be divided in to two groups to participate in either a focus group or an individual interview. Focus group participants will be grouped by level of participation (determined by website points; 150 (high), 10-100 (medium), = < 10 (low)), and purposively sampled to aim for a mix of sex, with participants from a variety of tutor groups. This sampling strategy will enable us to obtain views and actual experiences from a diverse cohort of participants. Each focus group (1-2 per school) will comprise 2–5 individuals (dependent on school scheduling and communication).

Individual interviews will include two shy and inactive individuals per school. These individuals will be purposely sampled based on T1 (baseline) questionnaire data; specifically, shyness and sociability data, provided by two 5-item measures from EAS temperament scale [24], included in the T1 questionnaire data. Physical activity will be determined by baseline self-reported youth physical activity questionnaire (YPAQ) questionnaire data. Frequency will be calculated as the summed sessions/week of all reported activities. Students who exhibit greater degrees of shyness and sociability (lowest scoring tertile) and those who participate in the least physical activity (lowest scoring tertile) will be invited to participate in an individual interview. Interviews will focus on the perceptions of the GoActive intervention from diverse student perspectives, including those with high shyness and low physical activity, who may be less likely to engage with physical activity promotion interventions.

Mentors will be informed of the focus group interviews upon their involvement in the intervention. All mentors at each intervention school will be invited to take part in the focus group interview. The number of focus groups with mentors will range from 1 to 3 per school (dependent on the number of mentors, recruitment and size of school), and comprise 4-8 individuals.

#### Focus group interviews

Semi-structured interactive focus group interviews with Year 9 students will be conducted after the facilitated intervention phase (first six weeks), providing participants with the opportunity to articulate their GoActive experience.

Mentor focus group interviews will explore participants’ perceptions of the GoActive intervention, the experiences of those directly involved with the intervention, and their views on what worked or did not work with regard to commencing the intervention. Questions will relate to the barriers and facilitators for implementation, whether or not the mentors see a future for the intervention in the school and why, and discussion about what would be required in the future to ensure that they continue with the intervention.

An interview guide, developed for both students and mentors (Additional files 2 and 3), and will be updated as new issues and themes emerge throughout each focus group; participants were encouraged to discuss additional issues.

#### Individual interviews

Individual interviews with a purposive sample of shy and inactive participants at all intervention schools will provide a deeper understanding of their views and experiences, and barriers/facilitators to their participation (a one-to-one interview may be more comfortable for these individuals). Interviews will be semi-structured, using a flexible interview guide, which will be piloted in the first few interviews, and adapted where necessary.

All intervention school contact teachers and local authority-funded facilitators will be invited to participate in an individual interview. For contact teachers, the individual interviews will explore the reasons for the adoption of the GoActive intervention, the facilitation of the intervention, the reception of the school teachers, as well as their perceptions of the collaboration between the GoActive team and their participating school. Furthermore, the interview will provide an opportunity to discuss the feasibility of future implementation of the intervention.

We will aim to individually interview all facilitators. Emphasis will be placed on all aspects of intervention delivery, feasibility and acceptance. Interviews will include discussions of barriers and facilitators for implementation, and capture contextual issues (such as timing, availability of resources, or facilities) that may have shaped the delivery of the intervention.

#### Mentor and facilitator logbooks

Mentor and facilitator GoActive written logbooks (housed on the GoActive website, and completed electronically) will be used to assess frequency of the intervention delivery. The mentors and facilitators will be responsible for completing weekly logs online, specifying the intervention activities chosen by each class, the facilitation support they provide, plus any other comments or information deemed valuable. Members of the study team will review the logs and send reminders for completion of logs where necessary.

## Data analysis

Quantitative, and open qualitative responses from questionnaires, as well as mentor and facilitator logs will be imported into Stata [25]. Process evaluation data will be analysed independently of the primary outcome data.

### Quantitative data analysis

Questionnaire data collected from all participating Year 9 students (control and intervention), mentors, teachers, and facilitators will be analysed and compared within groups. Descriptive statistics (comparisons of means, medians or % as appropriate) will allow the research team to assess intervention delivery, provide information about the differential implementation rates of the intervention’s essential functions, fidelity, enjoyment and satisfiability from all questionnaire data. Differences between intervention and control participants will be assessed statistically with linear or logistic regression, accounting for school clustering. In addition to questionnaire data, website analytics of the GoActive website will be used to generate descriptive statistics to explore fidelity, and dose received of the intervention.

### Qualitative data analysis

All interviews will be audio-recorded and transcribed verbatim. The transcripts will be checked for accuracy against the sound files and corrections will be made as appropriate. Any identifiable comments will be anonymised prior to transcripts being imported into NVivo. All qualitative data, including interview transcriptions, field notes from observations, emails, free-text from questionnaires, and mentor and facilitator logs, will be analysed using thematic content analysis, facilitated by QSR NVivo [26]. Adaptations and rationale behind all reported changes to the intervention will also be listed, analysed and summarised.

Analysis will follow a six phase model [27]. Initially, the mixed methods researcher will code 10% of transcripts allowing them to become reacquainted or familiarised with the data. This early coding will be inductive and deductive based on the interview guide, incorporating emerging themes as well as topics presented a priori in the interview guide. This subset of transcripts will be double coded by an independent coder, who will develop their own inductive coding scheme. Codes will be discussed by the two coders, and coding schemes will be refined and amended via an iterative process prior to the mixed method researcher continuing further coding of the transcripts.

Interim themes will be discussed by members of the research team to reach consensus. Initially, observational field notes, focus group, and individual interview data will be separated. Similar coding schemes will be used for the qualitative data collected with different sub groups, but will remain open to account for any required additions (e.g. where one set of results uncovers a theme not covered by other results). The coding schemes from each data set will be reviewed to determine if particular perspectives were weighted differently from others. Given the range of qualitative data sets used, qualitative data sets will then be used comparatively to triangulate the data to address completeness, convergence, and dissonance of key themes.

### Mixed methods analysis and integration

**Fig. 5:**
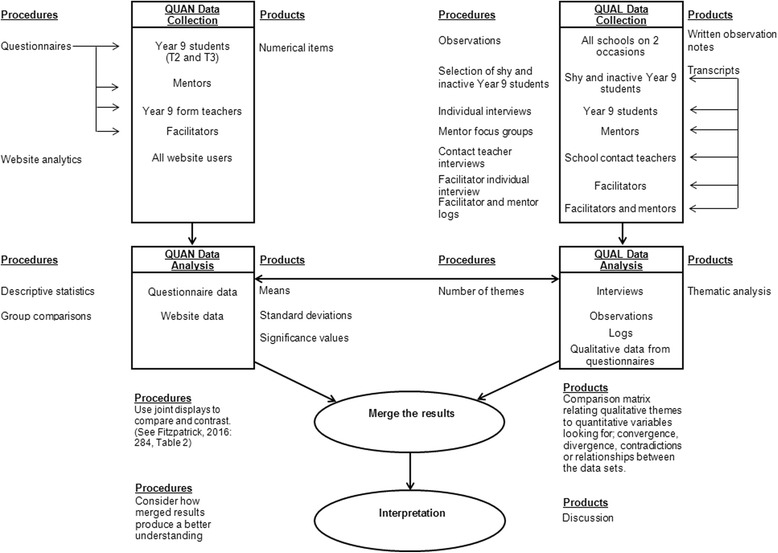
Procedural diagram for the GoActive mixed methods process evaluation

Effective integration is dependent upon four key components: level of integration, priority of quantitative and qualitative strands, timing, and where and how the quantitative and qualitative strands will be combined [19]. Within the GoActive process evaluation, the timing of the data collection will be concurrent and the level of integration will be interactive. Each of the two strands, quantitative and quantitative, will be distinct, and data will be collected at similar time points. Qualitative and quantitative data will be analysed separately and then mixed during analysis. Both the quantitative and qualitative strands have equal emphasis as both will play an equally important role in addressing the process evaluation research questions. A diagram of the process evaluation analytical procedure is provided in Fig. 5.

Coding of qualitative data and initial qualitative analysis, and generation of descriptive statistics will occur concurrently. Descriptive statistics will provide the mixed methods researcher direction upon returning to qualitative data analysis through the establishment of refined questions that will guide further qualitative analysis. This approach to integrating data is referred to as ‘following a thread’ [28]. For example, a key theme may arise from the quantitative data, which could be further explored or explained with quantitative findings. The same will also arise from the qualitative data analysis to the quantitative data.

The mixed methods researcher will then compare and integrate findings from the different data sources. Data integration will be guided by triangulation protocol [28], and will bind together data sets to conduct cross data comparison and convergence, creating a matrix. The mixed methods researcher will then be able to assess degrees of agreement, complementarity, and dissonance across the datasets, and identify areas of ‘silence’ (i.e. where a theme or finding arises from one data set and not another) [28]. The development of the triangulation protocol strengthens the researcher’s understanding of themes, creating meta-themes, cutting across findings from different methods [29]. Findings will then be summarised by producing a convergence coding matrix that covers all GoActive data (see for example, [30, 31]).

The finalised convergence matrix will display a synthesis of findings from the different data sources. The matrix will be used to highlight key mechanisms that are essential for the success of GoActive, as well as any implementation failures, or implementation difficulties, and will be used to explain the outcomes of the trial.

## Discussion

This paper describes the design of a mixed methods process evaluation embedded within the GoActive evaluation, a school based trial assessing the effectiveness of a complex intervention. The process evaluation is designed to investigate the implementation of the GoActive intervention, and provide a holistic understanding of the intervention’s outcomes. By publishing the protocol for this process evaluation, we make our methodological choices explicit and transparent, add to the literature of process evaluation protocols and mixed methods designs, and highlight the importance of process evaluation in public health and physical activity interventions. Although our process evaluation has been detailed, we recognise the importance of retaining flexibility to examine unexpected topics/events that arise. A mixed methods approach allows this, specifically with the exploratory and flexible qualitative methods [3].

The MRC guidance on complex intervention trials calls for standardisation but also recognises the need to ‘describe the constant and variable components of a replicable intervention’ [32]. In this light, process evaluations of public health interventions allow exploration of intervention delivery across diverse settings to understand how interventions are adapted to a local context. Given the variability of context across school settings, it is to be expected that different intervention essential functions will resonate more with some sites than others, reducing their intervention fidelity. For this intervention, fidelity, or meeting an intervention’s essential functions, has been typically identified as website use, form groups having mentors, mentors and schools utilizing facilitators etc. However, it may be unrealistic to expect *standardised* delivery and implementation of complex interventions across varying contexts [33]. As such, future research could explore whether fidelity to interventions of this complex nature is a predictor of success. Process evaluation that is inclusive of the investigation of context, as well as the function and process of the intervention across different settings, may help to understand overall effectiveness [33].

### Strengths and limitations

Similar to other published process evaluations [4], we have had to make some compromises due to time, resource constraints, and due to the general nature of the secondary school environment (e.g. time spent out of class, timetabling, exams). For example, the number of observations of the GoActive sessions will be kept to a maximum of two per school. We recognise that a larger number of observations of intervention sessions may enable a greater understanding of intervention delivery and acceptability, as well as a greater understanding of group interaction, and school context.

The role of the mixed methods researcher may also have an impact on reactivity, and may inhibit feedback about the trial. All participants will be made aware of the mixed methods researcher’s role in the research team; however, participants may assume that the mixed methods researcher will be involved in decisions about design and implementation. This may have an impact on what is disclosed within interviews and general conversations with participants.

Despite these limitations, the GoActive process evaluation has the potential to provide valuable insight into which components of the intervention being delivered worked best (and what did not), as well as develop an understanding of how and why the intervention had the effects it did (or did not) in each school, and to draw out implications for improving future school based physical activity interventions. As well as strengthening interpretation of results from the ongoing effectiveness evaluation, the process evaluation results will contribute to the developing understanding and overall body of work on the value of process evaluation in complex physical activity trials.

### Trial status

Currently, the trial is ongoing and recruitment of participants for process evaluation continues.

## Additional files

Additional file 1: SPIRIT check list. (DOC 145 kb)

Additional file 2: Focus group interview guide (mentors). (DOCX 18 kb)

Additional file 3: Focus group interview guide (students). (DOCX 19 kb)

## Abbreviations

MRC: Medical Research Council
MVPA: Moderate to vigorous physical activity
RCT: Randomised controlled trial
SPIRIT: Standardized Protocol Items: Recommendations for Interventional Trials

## References

[1] Century J, Rudnick M, Freeman C (2010). A Framework for Measuring Fidelity of Implementation: A Foundation for Shared Language and Accumulation of Knowledge. Am J Eval.

[2] Grant A, Treweek S, Dreischulte T, Foy R, Guthrie B. Process evaluations for cluster-randomised trials of complex interventions: A proposed framework for design and reporting. Trials. 2013;14. 10.1186/1745-6215-14-15 PMC3600672 23311722

[3] Craig P, Dieppe P, Macintyre S, Michie S, Nazareth I, Petticrew M, Medical Research Council G (2008). Developing and evaluating complex interventions: the new Medical Research Council guidance. BMJ.

[4] van de Glind I, Bunn C, Gray CM, Hunt K, Andersen E, Jelsma J, Morgan H, Pereira H, Roberts G, Rooksby J, et al. The intervention process in the European Fans in Training (EuroFIT) trial: A mixed method protocol for evaluation. Trials. 2017;18. 10.1186/s13063-017-2095-0 PMC5531072 28750673

[5] Masterson-Algar P, Burton CR, Brady MC, Nicoll A, Clarke CE, Rick C, Hughes M, Au P, Smith CH, Sackley CM. The PD COMM trial: A protocol for the process evaluation of a randomised trial assessing the effectiveness of two types of SLT for people with Parkinson’s disease. Trials. 2017;18. 10.1186/s13063-017-2130-1 PMC5576370 28851443

[6] Grant A, Dreischulte T, Treweek S, Guthrie B. Study protocol of a mixed-methods evaluation of a cluster randomized trial to improve the safety of NSAID and antiplatelet prescribing: Data-driven quality improvement in primary care. Trials. 2012;13. 10.1186/1745-6215-13-154 PMC3502604 22929598

[7] Ellard DR, Taylor SJ, Parsons S, Thorogood M. The OPERA trial: A protocol for the process evaluation of randomised trial of an exercise intervention for older people in residential nursing accommodation. Trials. 2011;12. 10.1186/1745-6215-12-28 PMC3037884 21288341

[8] Mann C, Shaw A, Guthrie B, Wye L, Man MS, Hollinghurst S, Brookes S, Bower P, Mercer S, Salisbury C (2016). Protocol for a process evaluation of a cluster randomised controlled trial to improve management of multimorbidity in general practice: the 3D study. BMJ Open.

[9] Griffin TL, Pallan MJ, Clarke JL, Lancashire ER, Lyon A, Parry JM, Adab P. Process evaluation design in a cluster randomised controlled childhood obesity prevention trial. the WAVES study. Int J Behav Nutr Phys Act. 2014;11. 10.1186/s12966-014-0112-1 PMC4172839 25212062

[10] Campbell R, Rawlins E, Wells S, Kipping RR, Chittleborough CR, Peters TJ, Lawlor DA, Jago R. Intervention fidelity in a school-based diet and physical activity intervention in the UK: Active for Life Year 5. Int J Behav Nutr Phys Act. 2015;12. 10.1186/s12966-015-0300-7 PMC4642771 26559131

[11] Griffin TL, Clarke JL, Lancashire ER, Pallan MJ. Process evaluation results of a cluster randomised controlled childhood obesity prevention trial: the WAVES study. BMC Public Health. 2017;17. 10.1186/s12889-017-4690-0 PMC5576245 28851329

[12] Edwardson CL, Harrington DM, Yates T, Bodicoat DH, Khunti K, Gorely T, Sherar LB, Edwards RT, Wright C, Harrington K, Davies MJ. A cluster randomised controlled trial to investigate the effectiveness and cost effectiveness of the ‘Girls Active’ intervention: A study protocol. BMC Public Health. 2015;15. 10.1186/s12889-015-1886-z PMC4453020 26036965

[13] Sebire SJ, Edwards MJ, Campbell R, Jago R, Kipping R, Banfield K, Tomkinson K, Garfield K, Lyons RA, Simon J, et al. Protocol for a feasibility cluster randomised controlled trial of a peer-led school-based intervention to increase the physical activity of adolescent girls (PLAN-A). Pilot and Feasibility Stud. 2016;2. 10.1186/s40814-015-0045-8 PMC4770840 27966675

[14] Brown HE, Whittle F, Jong ST, Croxson C, Sharp SJ, Wilkinson P, Wilson EC, van Sluijs EM, Vignoles A, Corder K. A cluster randomised controlled trial to evaluate the effectiveness and costeffectiveness of the GoActive intervention to increase physical activity among 13–14-year-old adolescents. BMJ Open. 2017;0:e014419. 10.1136/bmjopen-2016-014419 PMC5623411 28963278

[15] Corder K, Schiff A, Kesten JM, van Sluijs EM (2015). Development of a universal approach to increase physical activity among adolescents: the GoActive intervention. BMJ Open.

[16] Moore G, Audrey S, Barker M, Bond L, Bonell C, Hardeman W, Moore L, O’Cathain A, Tinati T, Wight D, Baird J (2014). Process evaluation of complex interventions: UK Medical Research Council (MRC) guidance.

[17] Moore GF, Audrey S, Barker M, Bond L, Bonell C, Hardeman W, Moore L, O’Cathain A, Tinati T, Wight D, Baird J (2015). Process evaluation of complex interventions: Medical Research Council guidance. BMJ.

[18] Saunders RP, Evans MH, Joshi P (2005). Developing a process-evaluation plan for assessing health promotion program implementation: a how-to guide. Health Promot Pract.

[19] Creswell JW, Plano Clark V (2011). Designing and conducting mixed methods research.

[20] Standardized Protocol Items: Recommendations for Interventional Trials (SPIRIT). [http://www.spirit-statement.org]. Accessed 5 Jan 2018.

[21] Salmon J, Jorna M, Hume C, Arundell L, Chahine N, Tienstra M, Crawford D (2011). A translational research intervention to reduce screen behaviours and promote physical activity among children: Switch-2-Activity. Health Promot Int.

[22] Creswell JW (2013). Qualitative Inquiry & Research Design: Choosing Among Five Approaches.

[23] Wragg EC (1994). An introduction to classroom observation.

[24] Buss AH, Plomin R (1984). Temperament. Early Developing Personality Traits.

[25] StataCorp (2015). Stata Statistical Software: Release 14.

[26] NVivo qualitative data analysis Software; QSR International Pty Ltd. Version 11. 2015. http://www.qsrinternational.com/nvivo/support-overview/faqs/how-do-i-cite-nvivo-for-mac,-nvivo-11-for-windows.

[27] Braun V, Clarke V (2006). Using thematic analysis in psychology. Qual Res Psychol.

[28] O’Cathain A, Murphy E, Nicholl J. Three techniques for integrating data in mixed methods studies. BMJ. 2010;341:c4587. 10.1136/bmj.c4587 20851841

[29] Farmer T, Robinson K, Elliott SJ, Eyles J (2006). Developing and implementing a triangulation protocol for qualitative health research. Qual Health Res.

[30] Fitzpatrick KR (2014). Points of Convergence in Music Education. J Mixed Methods Res.

[31] Wittink MN, Barg FK, Gallo JJ (2006). Unwritten rules of talking to doctors about depression: integrating qualitative and quantitative methods. Ann Fam Med.

[32] Medical Research Council (2000). A framework for the development and evaluation of RCTs for complex interventions to improve health.

[33] Hawe P, Sheill A, Riley T. Complex interventions: how “out of control” can a randomised controlled trial be? BMJ. 2004;328:1561. 10.1136/bmj.328.7455.1561 PMC437159 15217878

