# Supplementary material for: GoActive: a protocol for the mixed methods process evaluation of a school-based physical activity promotion programme for 13–14year old adolescents
Source: Trials. 2018 May 21;19:282. doi: 10.1186/s13063-018-2661-0 (PMC5963130; doi:10.1186/s13063-018-2661-0)
Supplement: Supplementary file 2 — Focus group interview guide (mentors). (DOCX 18 kb) [file 13063_2018_2661_MOESM2_ESM.docx]

**Additional file 2:**

**FOCUS GROUP INTERVIEW GUIDE: MENTORS**

**Focus group Introduction:**

Thank you for coming along today.

My name is XXXX and I work at the Centre for Diet and Activity Research (CEDAR), within a unit run by MRC Epidemiology, on a project called GoActive (which you may have heard about!).

Today I will be inviting you to participate in something called a focus group, which is like a discussion.

In this discussion, we’d like you to say exactly what you are thinking - there are no right or wrong answers! I will ask questions what about your thoughts about GoActive, and your experiences as mentors within the programme. It is important that you are honest and tell me what you think, even if it is different from what the other people are saying.

**Consent:**

At the start of GoActive, you might remember that we gave you an information sheet about GoActive. Your parents/careers have provided consent for you to participate in GoActive as a mentor, and at the start of your role as mentor within GoActive, you indicated that you were happy to participate in a focus group discussion. Participating in the discussion is completely up to you. If you are happy to take part then please stay, and if you do not want to take part, feel free to leave the room and return to class. You are free to leave the discussion whenever you would like.

**Confidentiality and group rules:**

I will be using a voice recorder to record our discussion so that I can remember what we have said. Next, we will get someone to type out our entire discussion so that I can read through it. We won’t use your names; instead we will say that ‘participant said this’, or ‘students said this’.

___________________________________

Please try and wait until someone else has finished talking so that I can hear what everyone has to say. I’d love to hear all of your thoughts on every topic we discuss! If you do not wish to answer a question, that is absolutely fine; feel free to say ‘pass’.

___________________________________

During the discussion some of the other discussion group members may say something you disagree with. You do not have to agree, but it is important to remember to respect each other’s views and opinions. Please keep private what other people say in today’s discussion.

___________________________________

Are there any questions?

**GROUP INTRODUCTIONS**

1. We will go round the group and I would like you to say your name, and what your favourite subject is at school, and what your favourite things to do outside of school are.
2. Are you planning to select physical education for GCSE?

Probe: Discuss choices in more detail

- How long have you been participating in ______(certain activities)?
- Do you compete in __________(activity) at a high level?

**GOACTIVE RELATED QUESTIONS**

1. How were you selected to be mentors?

Probe:

- - Did you teacher choose you? Which teacher?
  - How were you assigned to classes?

1. What did you understand your role to be as a mentor?
2. Take me through what you would do when preparing for a normal GoActive session?
3. What does your designated class look like when participating in GoActive?

Probe:

- - What kind of activities do you do?
  - Did you make any suggestions about activities?
  - Is everyone involved? Who isn’t?
  - Was it competitive?

1. What is going well in GoActive at this point?
   - Is there anything that you are particularly enjoying or anything that you dislike?
2. Can you tell me about how your teacher is involved in GoActive?

Probe

- - What is their role?
  - What do you think they should be doing?

1. Can you tell me about the Year 9 class leaders that work with you?

Probe

- - What is their role?
  - What do you think they should be doing?
  - Who decides on who they are?

1. Can you tell me about your meetings with the local authority funded council facilitator?

Probe

- - What is their role?
  - What do you think they should be doing?
  - How frequent are your meetings?
  - What do you talk about in your meetings?
  - Do you think their role is useful?

1. Do you have any suggestions on how GoActive can improve?
2. If you were given the task to talk to a potential mentor for next year, what would you say to them?

Probe

- - Would you participate as a mentor in GoActive again? Why/why not?

**CONCLUSION**

That’s all the questions we have for you today. Your responses have helped us a lot! Is there anything else you’d like to tell us about the things we talked about today?

Do you have any questions for me?

Thank you very much for your time and attention. We appreciate you sharing your thoughts and experiences with us.
