# Supplementary material for: GoActive: a protocol for the mixed methods process evaluation of a school-based physical activity promotion programme for 13–14year old adolescents
Source: Trials. 2018 May 21;19:282. doi: 10.1186/s13063-018-2661-0 (PMC5963130; doi:10.1186/s13063-018-2661-0)
Supplement: Supplementary file 3 — Focus group interview guide (students). (DOCX 19 kb) [file 13063_2018_2661_MOESM3_ESM.docx]

**Additional file 3:**

**FOCUS GROUP INTERVIEW GUIDE: YEAR 9 STUDENTS**

**Focus group Introduction:**

Thank you for coming along today.

My name is XXXX and I work at the Centre for Diet and Activity Research (CEDAR), within a unit run by MRC Epidemiology, on a project called GoActive (which you may have heard about!).

Today I will be inviting you to participate in something called a focus group, which is like a discussion.

In this discussion, we’d like you to say exactly what you are thinking - there are no right or wrong answers! I will ask questions what about your thoughts about GoActive, and your experiences with the programme. It is important that you are honest and tell me what you think, even if it is different from what the other people are saying.

**Consent:**

At the start of the GoActive, you might remember that we gave you an information sheet about GoActive. Your parents/careers have provided consent for you to participate in GoActive, and at the start of GoActive you indicated that you were happy to participate in a focus group discussion. In the last GoActive questionnaire that you filled out, we noticed that you ticked a box which indicated you would be happy to take part in the discussion today. We sent another letter home that looks like this (shows letter). Participating in the discussion is completely up to you. If you are happy to take part then please stay, and if you do not want to take part, feel free to leave the room and return to class. You are free to leave the discussion whenever you would like.

**Confidentiality and group rules:**

I will be using a voice recorder to record our discussion so that I can remember what we have said. Next, we will get someone to type out our entire discussion so that I can read through it. We won’t use your names; instead we will say that ‘participant said this’, or ‘students said this’.

___________________________________

Please try and wait until someone else has finished talking so that I can hear what everyone has to say. I’d love to hear all of your thoughts on every topic we discuss! If you do not wish to answer a question, that is absolutely fine; feel free to say ‘pass’.

___________________________________

During the discussion some of the other discussion group members may say something you disagree with. You do not have to agree, but it is important to remember to respect each other’s views and opinions. Please keep private what other people say in today’s discussion.

___________________________________

Are there any questions?

**GROUP INTRODUCTIONS**

1. We will go round the group and I would like you to say your name, and what your favourite subject is at school, and what your favourite things to do outside of school are.

Probe: Discuss choices in more detail

- How long have you been participating in ______(certain activities)?
- Do you compete in __________(activity) at a high level?
- Is it a place where you have met many friends?

**GENERAL PHYSICAL ACTIVITY QUESTIONS**

1. What are your thoughts about being active?

Probe:

- Do you enjoy it? Do you think it is hard/easy?
- When do you think you are being physically active?
- You say you enjoy ___________(activity) – do you think you are being active? Why?

**GOACTIVE RELATED QUESTIONS**

**Class related:**

1. Have you heard of GoActive? What can you tell me? What do you think it is about?
2. How has your class been involved in GoActive?

Probe: If no….

- - What would have to happen for you to participate in GoActive?
  - If you were me, and you were trying to get your friends active, what would you do?/// If we were starting from scratch what should we do to get adolescents involved in physical activity?

1. What does your class look like when participating in GoActive?

Probe:

- - What kind of activities do you do?
  - Did you make any suggestions about activities?
  - Is everyone involved? Who isn’t?
  - Was it competitive?
  - When you are split into different teams, have you spoken with people you don’t normally talk with from your class?

1. How do you normally spend tutor time? How do you find doing GoActive in tutor time?
2. What is going well in GoActive at this point?
   - Is there anything that you are particularly enjoying or anything that you dislike?

**Role related (leader, mentor and teacher):**

1. Can you tell me about your class leaders?

Probe

- - What is their role?
  - What do you think they should be doing?
  - Who decides on who they are?

1. Can you tell me about your mentors?

Probe

- - What is their role?
  - What do you think they should be doing?

1. Can you tell me about how your teacher is involved in GoActive?

Probe

- - What is their role?
  - What do you think they should be doing?

**Website related:**

1. How have you found the GoActive website?
2. What do you think of having to enter points online for each GoActive activity?
   - Have you entered points on to your account?
   - Do you think everyone is honest when entering their GoActive points online? Why/why not?
   - Is the points system competitive? Why/why not?

**Class related:**

1. (if they do) Why do you keep participating in GoActive?
2. Do you have any suggestions on how GoActive can improve?

**CONCLUSION**

That’s all the questions we have for you today. Your responses have helped us a lot! Is there anything else you’d like to tell us about the things we talked about today?

Do you have any questions for me?

Thank you very much for your time and attention. We appreciate you sharing your thoughts and experiences with us.
